# Ten simple rules for writing a cover letter to accompany a job application for an academic position

**DOI:** 10.1371/journal.pcbi.1006132

**Published:** 2018-05-31

**Authors:** Lubomir Tomaska, Jozef Nosek

**Affiliations:** 1 Department of Genetics, Faculty of Natural Sciences, Comenius University in Bratislava, Bratislava, Slovakia; 2 Department of Biochemistry, Faculty of Natural Sciences, Comenius University in Bratislava, Bratislava, Slovakia; Whitehead Institute, UNITED STATES

## Introduction

It is becoming a difficult task to find an academic position that is best suited for one’s capabilities and preferences. In an extremely competitive environment [1], there are tens of applicants (and often more) per a single position. As a result, the hiring committees judge the quality of the candidates based on numerous criteria, including previous achievements listed in their CVs; recommendation letters from their instructors, supervisors, or peers; technical and presentation skills; and research plans. In many cases, the first encounter with the applicants is mediated by their cover letter. If well crafted, the letter can simultaneously act as an introduction, a first-stage filter, and a cogent, compelling argument for one’s candidacy (e.g., [2–6]). On the other hand, a generic, boring, uninspiring cover letter full of typos will increase the probability of dismissal of the application, oftentimes irreversibly blocking the applicant’s entry to a potential dream position. The list of rules below should be helpful in composing a cover letter that will serve as a catalyst for pushing the application into the next stages of evaluation. The provided rules are specifically designed for job applications for an academic position (e.g., PhD student, postdoc, lecturer, faculty member). Although most also apply for other types of jobs (e.g., in industry), these may have specific requirements that need to be taken into account.

## Rule 1: Before starting the application, make a list of pros and cons for the position

On 11 November 1838, Charles Darwin proposed to his cousin, Emma Wedgwood, and wrote in his diary, “The day of days!” What followed (beginning 29 January 1839) were 43 years of happy marriage, underlining the fact that Darwin’s proposal and Ms. Wedgwood’s acceptance were both correct decisions. Perhaps what is not so widely known is that Darwin, in the months immediately preceding his engagement, had written two notes weighing up the pros and cons of marriage [7]. Following Darwin’s example, before starting to compose an application letter, it should prove helpful to summarize the reasons in favor of (and also against) applying for the position. Perform detailed research of the available positions that may suit you. Go beyond the information provided by job ads and institution websites; consult mentors, advisers, and colleagues. Start writing the cover letter only if the pros of application outweigh the cons. This will help you to compose a text in an enthusiastic style.

## Rule 2: Remember KISS—Keep It Short and Simple

This is one of the key concepts in effective speaking and writing. For a cover letter, if not required otherwise by specific formal requirements, two pages are the maximum. Address the letter to a named person. A short introduction serves as a “handle,” i.e., it should get attention from the committee members [2]. Then you can proceed to a brief and clear summary of your most important—and relevant—qualifications. The balance between your research, teaching, and administration (or other) skills depends on the nature of the position. Next, explain what attracts you to the position and how it fits into your career plan. To be concise, stay focused. Anything less than a sharp focus and your readers will quickly lose interest and move on to the next application. Do not duplicate your CV. Rather, emphasize what does not get covered or rise to the surface in your CV or résumé. Make sure your cover letter is consistent with your CV. In the case of a digital cover letter, you can provide active links to information that may be relevant such as your website or list of publications (e.g., as a link to ResearcherID, ORCID, or GoogleScholar). The closing of the letter is as important as its opening. Do not let it meander to an indefinite or weak last paragraph. End your letter decisively by including a statement expressing interest in an interview.

## Rule 3: Be original, nonconformist, and personal

Although it is useful to read cover letters of successful candidates, do not get too influenced by their style and content. Be yourself. Think of your cover letter as the opener to your application, similar to a cork that represents an entry to the contents of a wine bottle. Just as a cracked or rotten cork will discourage a user from pouring the contents of the bottle into a glass and evaluating all of its attributes, an uninspiring cover letter might prevent the recruiter from reading the full application and assessing your suitability for the position.

## Rule 4: Show motivation and sincere interest

If you are applying for a research position, try to address the following questions: why you are choosing this position; what is exciting about the projects performed in the laboratory; and what part of the project you would like to pursue. You need to show that you did not just read the titles of recent publications but that you are familiar with the methodology, experimental design, and analysis as they are performed by your prospective employer. This will help you suggest future experiments or research directions aimed at better understanding of corresponding phenomena. In case of a faculty position, explain why you plan to pursue your career at the given institution; indicate how you will benefit from the collaboration with the groups at the department and vice versa, and how you plan to obtain support for your research; describe your experience with supervising students and postdocs; and show motivation for teaching, if the position requires teaching-related activities [8].

## Rule 5: Provide an honest description of yourself

Provide a glimpse of your personality, possibly in the form of a story that highlights your specific characteristics. Pick a few adjectives that describe you most of the time, regardless of the situation. You may provide information about the path that led to your interest in a particular field. Be positive.

## Rule 6: Highlight your strengths

How will the lab or institution benefit from having you onboard? List your major achievements and technical skills. State explicitly how your abilities and interests align with the position. Expand on what makes you especially suitable or appealing for the specific position you are applying for. Explain which of your strengths may set you apart from other candidates. If your background does not exactly match all of the criteria that the employer seeks, spell out what you are willing to do to learn the specific skills that the hiring organization needs (e.g., taking a special course or training). Do not forget about your behavioral strengths, such as your ability to lead a project, to work as a team member, or to be an effective communicator [9].

## Rule 7: Do not recycle

If you are sending applications for several positions, do not use the same text and just change the name of the institution. It is important to tailor your letter to the position you are applying for. Every research group and every institution prides itself on certain characteristics that make it unique. Therefore, generic, template-like letters are prone to be identified. They can make you seem rushed, noncommittal and not particularly interested in the position advertised and are most likely to end up in the recycle bin.

## Rule 8: Avoid overstatements and false claims

Overstatements tend to be annoying, and false claims, when uncovered, result in immediate rejection. Do not make yourself look better or more qualified. Avoid pompous metaphors and clichés. Be honest and truthful. Do not exaggerate. Also, do not be unrealistic in what you could achieve should you be offered the position, i.e., do not list promises that you cannot keep. Remember that it is always better not to be accepted for a position than to run into troubles or conflicts while holding it.

## Rule 9: Do not underestimate the formal quality of the letter

If a letter reads well, looks good, and is devoid of spelling and grammatical errors, then the reader will have a tendency to associate those qualities with the candidate. If someone is unable to express him/herself without errors, that triggers an immediate rejection [5]. Proofreading for content, accuracy, and style is crucial. Here are some suggestions: Spell check and get a colleague or trusted personal contact to check spelling and readability, too. Automated spell checkers may not catch inappropriate usage of similarly spelled words or homonyms; be particularly careful about writing the recipient's and institution’s names correctly, check any dates and addresses you are referencing, avoid lists or bullet points, and avoid unusual or unreadable fonts.

## Rule 10: Plan ahead and do not rush

When you think the letter is in the best shape possible, try to put yourself in the position of the recruiter. To do so, set it aside for a couple of days. Such detachment will help you to see flaws that were not obvious at the time of writing. Format the letter even if it is being sent by email. Ask for references or recommendations in advance. Allocate sufficient time to finalize the letter. Note that different countries may have different application processes and that not everything indicated above will apply to every country. Therefore, have someone with knowledge of local customs review the letter as well. Finally, do not miss the deadlines.

## Conclusion

Although a well-written cover letter is only a small step towards a successful job application, it may provide an important advantage by sparking the interest of the hiring committee for that particular applicant. Even if it is not formally required, it should still be included with the application, as it can help to underline the applicant's qualities related to the position description and it may point to a particular section of a lengthy résumé where they can find more detailed information [5]. To put it metaphorically, a cover letter is a handle on the door to the application, and depending on its quality, the door can remain closed, or it can be opened.
